# Treating Severe Primary Hypothyroidism: Turning Growth Failure Into Rapidly Progressive Puberty

**DOI:** 10.7759/cureus.81615

**Published:** 2025-04-02

**Authors:** Antony Fu, Kashima Takemoto, Reiko Horikawa

**Affiliations:** 1 Paediatrics, The Chinese University of Hong Kong, Hong Kong, HKG; 2 Paediatrics and Adolescent Medicine, Princess Margaret Hospital, Hong Kong, HKG; 3 Paediatric Endocrinology, National Center for Child Health and Development, Tokyo, JPN; 4 Endocrinology and Metabolism, National Center for Child Health and Development, Tokyo, JPN

**Keywords:** children, gonadotropin-inhibiting hormones, hypothyroidism, rapidly progressive puberty, short stature

## Abstract

Growth failure and precocious or rapidly progressive puberty have been documented in several instances of untreated overt primary hypothyroidism. However, the sequential occurrence of these conditions within the same patient is less frequently observed. In this report, we present a case wherein growth failure, followed by rapidly progressive puberty, manifested following thyroxine replacement therapy. Notably, the etiology of the puberty onset in this case was centrally derived, distinguishing it from the more commonly reported Van Wyk-Grumbach syndrome. This case report provides a comprehensive summary of the aforementioned case, conducts a thorough review of relevant literature, and proposes a plausible mechanistic explanation for the observed association with gonadotropin-inhibiting hormones. The significance of maintaining vigilant thyroid function screening to detect non-specific or atypical presentations of hypothyroidism is also underscored.

## Introduction

Primary hypothyroidism stands as one of the most prevalent endocrine disorders affecting the pediatric population. Among its various etiologies, autoimmune thyroiditis emerges as the leading cause of acquired hypothyroidism in children and adolescents [1]. The influence of thyroid hormones (THs) on growth promotion is well-established. While instances of hypothyroidism rarely coincide with the occurrence of sexual precocity, such instances are typically not rooted in central origins. This report details the case of a seemingly healthy adolescent female who first presented with markedly elevated levels of thyroid-stimulating hormone (TSH) and diminished free thyroxine (FT4) levels, observed during the assessment of non-specific symptoms. It is noteworthy to document the exceptional sequence of events: an initial three-year period of growth failure, followed by the abrupt onset of rapidly progressive puberty immediately after the initiation of thyroxine replacement therapy in the same patient. The role of hypothalamic gonadotropin-inhibitory hormone (GnIH) in the complex interactions between the hypothalamic-pituitary-thyroid (HPT) and hypothalamic-pituitary-gonadal (HPG) axes will also be explored.

## Case presentation

The subject of our study is a young girl of Japanese descent who came under the care of the Endocrinology Department at the age of 10 due to the diagnosis of primary hypothyroidism. Prior to her referral, the girl initially sought the expertise of a dermatologist concerning issues related to hair loss and dry skin. Subsequent laboratory analysis conducted by the dermatologist unveiled a severe case of primary hypothyroidism (as detailed in Table 1), leading to her referral to our department.

**Table 1:** Trend of thyroid functions of our case. TSH: thyroid-stimulating hormone; FT3: free triiodothyronine; FT4: free thyroxine

Date	4/4/2011	13/5/2011	17/6/2011	18/8/2011	14/12/2011	14/5/2012	13/3/2013	3/4/2014	2/4/2015	30/3/2020	Reference range
Age (years)	10					11	12	13	14	19	
TSH	991.0	5.68	0.32	0.78	2.662	4.119	2.10	0.1	0.355	1.751	0.61-4.23 mIU/L
FT3	0.90	5.82	6.01	4.04	3.84	3.61	3.3	4.72	3.62	3.35	2.52-4.06 pg/mL
FT4	0.32	1.43	1.57	1.04	1.17	1.11	1.67	1.42	1.21	1.44	0.68-1.26 ng/dL

**Figure 1:**
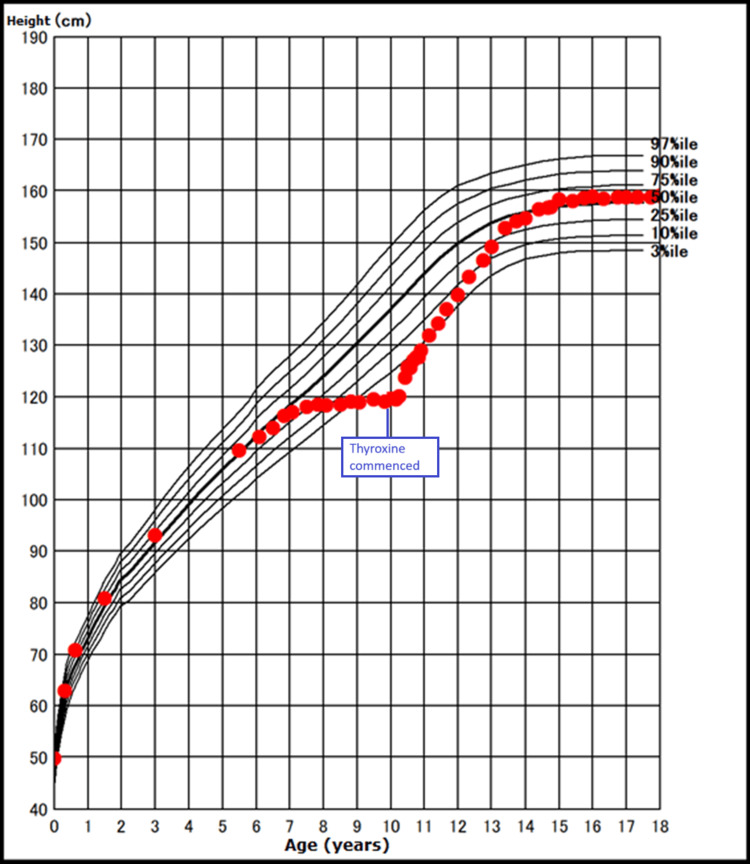
Height growth chart during the different growth phrases of our case.

**Figure 2:**
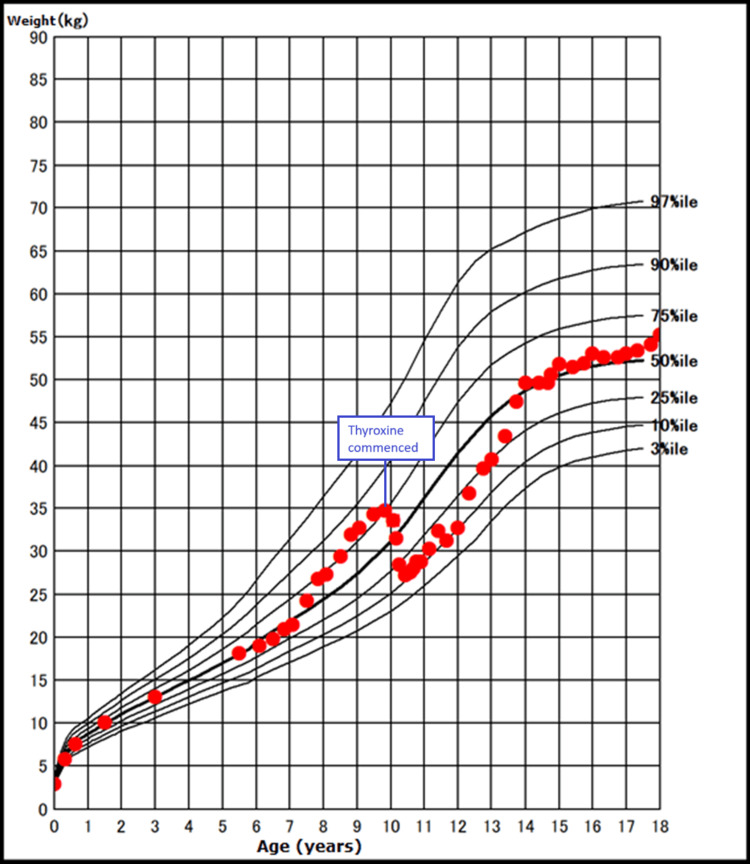
Weight growth chart during the different growth phrases of our case.

In retrospect, the parents of the patient noted a nearly halted progression in her height over a span of approximately three years preceding her current presentation. Throughout this period, her average annual growth velocity measured merely 0.7 cm/year. Intriguingly, this phase also saw accelerated weight gain compared to her prior trajectory (Figures 1-2).

Despite her diminished appetite, she did not exhibit additional symptoms commonly associated with hypothyroidism, such as reduced energy levels, constipation, or neck swelling. She had not yet reached menarche during this time.

She enjoyed good past health, except for a history of asthma. Her elder sister had type 1 diabetes and was currently on an insulin regimen. Otherwise, she did not have a family history of thyroid or puberty problems.

During the physical assessment, her stature was measured at 119.9 cm, reflecting a standard deviation (SD) score of -2.69 with regard to the norm. Her weight registered at 33.75 kg, corresponding to an SD score of +0.35, or the 73.6th percentile. Notably, there was an absence of goiter. She was in a prepubertal state, denoted by Tanner stage 1 breast development. The results of her systemic examination yielded no noteworthy findings.

Endocrine loading tests, including the thyrotropin-releasing hormone (TRH) stimulation test, luteinizing hormone-releasing hormone (LHRH) stimulation test, growth hormone-releasing hormone (GHRH) stimulation test, and corticotropin-releasing hormone (CRH) stimulation test, were conducted as illustrated in Table 2. They displayed biochemical confirmation of primary hypothyroidism, a borderline low post-stimulated growth hormone (GH) level (cutoff < 7 ug/L), an intact pituitary-adrenal axis, and prepubertal gonadotropin responses. Her prolactin level was raised, and her estradiol (E2) was undetectable. Meanwhile, her anti-thyroid peroxidase and anti-thyroglobulin antibodies were both elevated - over 600 IU/mL and 405 IU/mL, respectively.

**Table 2:** Endocrine loading tests (TRH stimulation test, LHRH stimulation test, GHRH stimulation test and CRH stimulation test) of our case at diagnosis. TSH: thyroid-stimulating hormone; ACTH: adrenocorticotropic hormone; GH: growth hormone; PRL: prolactin; LH: luteinizing hormone; FSH: follicle-stimulating hormone; TRH: thyrotropin-releasing hormone; LHRH: luteinizing hormone-releasing hormone; GHRH: growth hormone-releasing hormone; CRH: corticotropin-releasing hormone

	-30 min	0 min	15 min	30 min	60 min	90 min	120 min
TSH (mIU/L)	-	766.99	-	1961.71	2293.86	1927.84	1763.51
ACTH (pg/mL)	11.7	12.0	-	22.5	31.6	26.2	22.2
Cortisol (ug/dL)	12.1	12.0	-	25.1	28.3	25.7	25.9
GH (ug/L)	-	1.26	4.85	6.74	5.3	4.09	3.15
PRL (ng/mL)	-	24.78	-	99.13	68.0	54.46	45.53
LH (IU/L)	-	0.11	-	0.81	1.06	1.05	0.93
FSH (IU/L)	-	5.67	-	10.7	14.65	15.73	18.73

**Figure 3:**
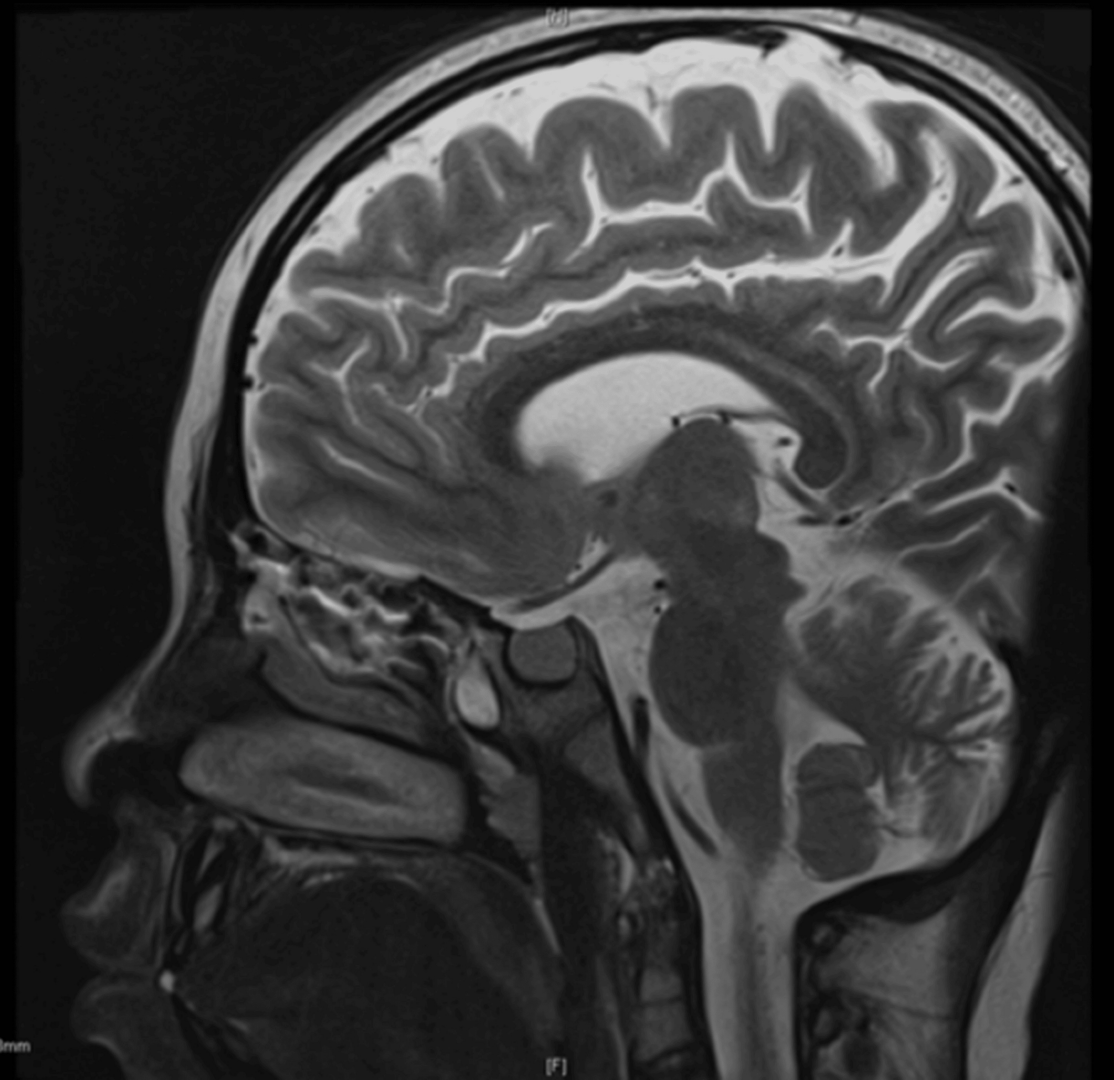
Sagittal plane of the MRI brain and pituitary of our case - showing the pituitary hyperplasia with otherwise no abnormal intensity. MRI: magnetic resonance imaging

At her chronological age of 10 years, her bone age was assessed as six years old, utilizing the standardized TW2 bone age estimation method specifically tailored for Japanese children and adolescents [2]. Evaluation through thyroid ultrasound revealed an enlarged gland characterized by irregular echo structures and heightened vascularization. Given the observed deviation in her growth trajectory, the diagnostic spectrum encompassed potential causes, including a brain tumor. Consequently, magnetic resonance imaging of her brain and pituitary was conducted, revealing mild pituitary gland enlargement devoid of abnormal signal intensity (Figure 3).

The administration of thyroxine replacement commenced at an initial dosage of 75 micrograms per day (equivalent to 2.2 micrograms per kilogram of body weight per day). In a month's time, restoration of euthyroidism was successfully achieved (Table 1).

Three months subsequent to the initiation of replacement therapy, a noticeable shift in her weight percentile was observed, aligning more closely with her initial baseline. Surprisingly, she exhibited a linear growth of 4 cm over the ensuing 4.2 months, reflecting an annual growth velocity of 11.5 cm/year. A pubertal assessment revealed an advancement to breast stage III, a stark difference from her prepubertal state observed four months earlier. Her hormonal profile indicated a pubertal status, with luteinizing hormone (LH) levels at 0.93 IU/L, follicle-stimulating hormone (FSH) at 8.31 IU/L, and estradiol at 44.9 pg/mL.

Thereafter, a decision was made to initiate GnRH analog therapy to suppress her rapidly advancing puberty in order to preserve her adult height. Regrettably, this intervention had to be prematurely halted after seven months due to the emergence of insurmountable side effects attributed to the injections. Simultaneously, the consideration of GH therapy was dismissed. This decision was influenced by her demonstrably favorable catch-up growth in height subsequent to thyroxine replacement. Moreover, her GH study during the thyroxine loading test lacked reliability, particularly given her non-euthyroid status at the time, which led to observed blunted responses of GH in some individuals with hypothyroidism [3].

Subsequently, she has consistently maintained a euthyroid state for the majority of this period. Her menstrual cycles have exhibited regularity since the onset of menarche at the age of 14. Of note, her weight and height have consistently tracked along the 50th to 75th percentiles, indicative of a trajectory toward adulthood.

## Discussion

THs play a pivotal role in the early stages of brain development, as well as in the processes of physical growth, bone development, and maturation during puberty [4]. In many respects, THs are viewed as tissue growth factors. THs potentiate GH stimulation of the synthesis and action of insulin-like growth factor 1 (IGF1), and the stimulation of the production of different growth factors (epidermal growth factor, erythropoietin, and nerve growth factor) [5].

Autoimmune hypothyroidism is the predominant acquired thyroid disorder in children and adolescents following birth [1]. The majority of these young individuals typically exhibit mild manifestations of the disease, often diagnosed and managed without significant complications. However, our presented case deviated from the expected norm, showcasing an atypical and less frequently encountered clinical trajectory.

In children, hypothyroidism can cause delayed skeletal development and bone age, with short stature, as illustrated by our case. The proliferative and hypertrophic zones of growth plates are decreased in height, and chondrocyte proliferation, chondrocyte hypertrophy, and vascular/bone cell invasion are affected in animals with hypothyroidism [6]. Meanwhile, their weight tends to increase.

The anterior pituitary shows an increase in thyrotroph cells in primary hypothyroidism. Hyperplasia or even adenoma formation may result from long-standing hypothyroidism, particularly hypothyroidism during infancy [7,8]. Similar to our case, enlargement of the pituitary fossa has been demonstrated.

Hypothyroidism is generally associated with delayed puberty [9], although precocious puberty can sometimes develop in untreated primary hypothyroidism - the Van Wyk-Grumbach syndrome (VWGS). The pathophysiology involves intricate interactions among multiple hypothalamic-pituitary hormonal pathways. Van Wyk and Grumbach suggested that the pituitary feedback mechanism exhibits hormonal overlap [10]. This may be attributed to similarities at the molecular level, as both TSH and gonadotropins are glycoproteins, and/or to a degree of non-specific regulation at the hypothalamic level [10].

TSH excess induced by TRH is probably the common stimulator of the FSH receptor. At the same time, hyperprolactinemia induced by TRH likely inhibits the pituitary gonadotrophic axis, particularly LH. As illustrated in our case, she had a borderline elevated spontaneous FSH level (5.67 IU/L), a low LH level (0.11 IU/L), and hyperprolactinemia at the time of presentation. Given that spontaneous FSH measurements are not useful in the diagnosis of central precocious puberty (CPP) [11], along with the fact that her spontaneous LH level was low and she was found to be prepubertal at the time of presentation, she was not considered CPP at that time.

Although the incidence of precocious puberty associated with severe primary hypothyroidism is as much as 24% [12], our case, however, was not VWGS. In the first place, she only developed pubertal signs after thyroxine replacement had taken place, when her TSH level was already back in the normal range. Furthermore, instead of pseudo-precocious puberty, as in VWGS, she experienced rapidly progressive puberty, as evidenced by the elevated gonadotropin levels after thyroxine replacement - i.e., central in origin.

One plausible explanation for the observed phenomenon within our case lies in the context of catch-up growth subsequent to thyroxine replacement, concomitant with the central onset of typical puberty. However, given the notably accelerated pace of her pubertal progression, it becomes imperative to explore alternative postulations.

We postulate that it might be related to the GnIH that interacts between the HPT and HPG axes. GnIH, also known as RFamide-related peptide (RFRP) in mammals and primates, is a recently discovered hypothalamic neuropeptide that actively inhibits gonadotropin synthesis. It was first identified in the cultured Japanese quail anterior pituitary [13] and later across various vertebrates, including mammals such as humans [14]. The inhibitory effect of GnIH on reproduction is mainly accomplished at hypothalamic-pituitary levels [15]. Direct inhibitory actions of GnIH on GnRH neurons in their neuronal activity, firing rate, and GnRH release have been demonstrated. It is also possible for GnIH to have an inhibitory role in the regulation of kisspeptin neurons [16]. It was demonstrated that increased hypothalamic GnIH and reduced Kiss1 messenger RNA (mRNA) in hypothyroid birds, and later in vertebrates including humans, resulted in reduced pituitary LH levels and decreased circulating E2 levels, leading to delayed puberty onset [17]. As in our case, when her euthyroidism was rapidly restored after thyroxine replacement, there was a significant decrease or withdrawal of GnIH neural function, and the pulsatile GnRH secretion increased, thereby inducing the central onset of puberty [18].

## Conclusions

In summary, this is an interesting case of stunted growth at presentation, but with notable improvement in growth velocity, together with the central onset of puberty upon correction of severe hypothyroidism. Instead of VWGS, her rapidly progressive puberty is more likely due to the withdrawal of hypothyroidism-induced GnIH actions following the restoration of thyroid function. Certainly, this case also demonstrates the potential consequences of diagnostic delays, culminating in the emergence of profound clinical sequelae. Furthermore, it underscores the imperative for a heightened index of suspicion when screening for thyroid function in the presence of atypical or nonspecific manifestations of hypothyroidism.
